# Translation of Cellular Protein Localization Using Convolutional Networks

**DOI:** 10.3389/fcell.2021.635231

**Published:** 2021-08-05

**Authors:** Kei Shigene, Yuta Hiasa, Yoshito Otake, Mazen Soufi, Suphamon Janewanthanakul, Tamako Nishimura, Yoshinobu Sato, Shiro Suetsugu

**Affiliations:** ^1^Division of Biological Science, Graduate School of Science and Technology, Nara Institute of Science and Technology, Ikoma, Japan; ^2^Division of Information Science, Nara Institute of Science and Technology, Ikoma, Japan; ^3^Data Science Center, Nara Institute of Science and Technology, Ikoma, Japan; ^4^Center for Digital Green-Innovation, Nara Institute of Science and Technology, Ikoma, Japan

**Keywords:** machine learning, Pix2pix, image conversion, WAVE2, lamellipodia

## Abstract

Protein localization in cells has been analyzed by fluorescent labeling using indirect immunofluorescence and fluorescent protein tagging. However, the relationships between the localization of different proteins had not been analyzed using artificial intelligence. Here, we applied convolutional networks for the prediction of localization of the cytoskeletal proteins from the localization of the other proteins. Lamellipodia are one of the actin-dependent subcellular structures involved in cell migration and are mainly generated by the Wiskott-Aldrich syndrome protein (WASP)-family verprolin homologous protein 2 (WAVE2) and the membrane remodeling I-BAR domain protein IRSp53. Focal adhesion is another actin-based structure that contains vinculin protein and promotes lamellipodia formation and cell migration. In contrast, microtubules are not directly related to actin filaments. The convolutional network was trained using images of actin filaments paired with WAVE2, IRSp53, vinculin, and microtubules. The generated images of WAVE2, IRSp53, and vinculin were highly similar to their real images. In contrast, the microtubule images generated from actin filament images were inferior without the generation of filamentous structures, suggesting that microscopic images of actin filaments provide more information about actin-related protein localization. Collectively, this study suggests that image translation by the convolutional network can predict the localization of functionally related proteins, and the convolutional network might be used to describe the relationships between the proteins by their localization.

## Introduction

Machine learning has achieved significant success in various fields, including the biomedical fields (Moen et al., 2019). Machine learning has been used to classify cellular images (Brent and Boucheron, 2018; Camacho et al., 2018; Moen et al., 2019). Among machine learning, convolutional networks, including U-net, have been shown to segment biomedical images, including cellular images (Ronneberger et al., 2015). After U-net, several applications of convolutional networks for the analysis of cellular images have been reported. Using bright-field cell images, radiation-resistant cells were distinguished from parental cells by machine learning (Toratani et al., 2018). Breast cancer cells treated with the anti-cancer agent paclitaxel were also distinguished from non-treated cells by machine learning (Kobayashi et al., 2017). Furthermore, the direction of cell migration was predicted using the sequences of cell images (Nishimoto et al., 2019). These results demonstrated that machine learning could extract information associated with cellular properties from images.

Machine learning has been applied not only in the classifications mentioned above but also in protein localization. For example, a method that is known as *in silico* labeling reportedly generated a putative stained image of a specific marker protein from bright-field cell images to identify the nuclei, neural cells, and live cells (Christiansen et al., 2018). Automatic segmentation of intracellular organelles such as the Golgi apparatus and endoplasmic reticulum from bright-field cell images was also achieved (Pärnamaa and Parts, 2017). However, the translation of protein localization to the localization of another protein has not been reported.

The generative adversarial network (GAN) is the method derived from the U-net, where the probability distribution model obtained through training with a number of paired images generates hypothetical images (Goodfellow et al., 2014). The GAN comprises two components: a generator and a discriminator; thus, it can generate high-quality images by competing between the generator and discriminator. Therefore, GAN generates a more similar image of A from an image of B than the U-net alone, after learning many paired images of A and B. For example, the GAN can reportedly generate an image of a “smiling” face from an image with a “non-smiling” face by learning many paired images of non-smiling and smiling faces (Sagawa and Hagiwara, 2018). Pix2pix is one of the major implementations of GAN in image-to-image translation problems (Isola et al., 2017). Pix2pix successfully generated many kinds of paired images, including a map from an aerial image, a color image from a black-and-white image, a label to a street scene, a biomedical image like that from MRI to the labels of the organs, and so on. In cell biology, pix2pix has been used to label cellular membranes and nuclei using images of their markers (Tsuda and Hotta, 2019), where label generation was performed by training with the image pairs of the labels indicating the membrane and nucleus (label images) and their actual images.

However, as far as we know, no report has demonstrated the application of convolutional networks including U-net and pix2pix to the generation of an image showing the cellular molecule localization at subcellular resolutions, i.e., the generation of images showing the localization of a protein from those of other proteins. We hypothesized that convolutional networks of U-net and pix2pix could be used to generate, i.e., to predict protein localization, depending on the relationships between the proteins.

**FIGURE 1:**
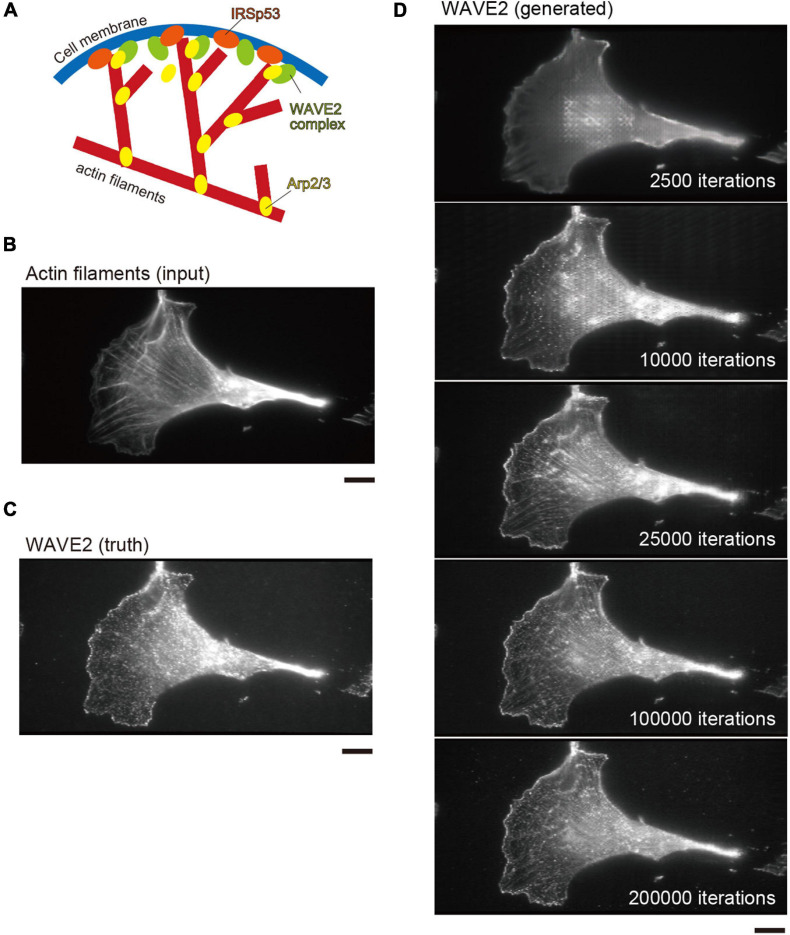
Lamellipodia and WAVE2 localization in Swiss 3T3 cells. **(A)** Schematic illustration of the configuration of actin filaments and WAVE2 localization at lamellipodia. Upon Rac1 activation, the WAVE2 in the protein complex is activated, leading to the activation of the Arp2/3 complex for branched actin filament formation. IRSp53 cooperates with WAVE2 for its activation by Rac1 at the plasma membrane. **(B)** Input image of actin filaments in Swiss 3T3 cells expressing the active form of Rac1. Actin filaments were stained by Rhodamine–phalloidin. Lamellipodia are fan-shaped structures formed at cell edges. **(C)** Actual WAVE2 image co-immunostained with panel **(B)**, showing accumulation at the edges of lamellipodia. **(D)** Progress of the WAVE2 image generation. Images are shown at every 2,500 iterations (1 epoch). The iteration number is shown in the images. Image generation starts with a gray image without any features. Scale bars, 10 μm.

Cells change their shapes based on the mitotic cycle, surrounding environment, and various other situations by altering the cytoskeleton, including actin filaments (Pollard and Borisy, 2003; Gunning et al., 2015). In cells, actin filaments further assemble into higher-order configurations, which are primarily determined by Rho-family small GTPases, including Cdc42, RhoA, and Rac1 (Hall, 1998; Takai et al., 2001). Among them, Rac1 induces actin filament branching through WASP-family verprolin homologous protein 2 (WAVE2) and the Arp2/3 complex (Bear et al., 1998; Machesky and Insall, 1998; Miki et al., 1998; Suetsugu et al., 1999b, 2003). The activation of Rac1 induces conformational changes in WAVE2 in the regulatory complex, consisting of Sra1/PIR121, WAVE2, Nap1, Abi1/2, and HSPC300/BRICK, leading to the activation of the Arp2/3 complex within the branched actin filaments (Innocenti et al., 2004; Suetsugu et al., 2006; Ismail et al., 2009; Chen et al., 2010; Figure 1A). IRSp53 is also involved in lamellipodia formation through WAVE2 (Miki et al., 2000; Suetsugu et al., 2006). Vinculin is a protein at focal adhesions, which are connected to actin filaments and promote lamellipodia formation (Ziegler et al., 2006). Lamellipodia are regarded as essential structures for cell migration, including cancer cell invasion and metastasis (Takenawa and Suetsugu, 2007; Ridley, 2011). Another cytoskeleton, the microtubule, is not directly related to the actin cytoskeleton. In this study, we translated the images of actin filaments of cells to those of WAVE2, IRSp53, vinculin, and microtubules using convolutional networks; then, we examined the quality of the translated images. The generated images of WAVE2, IRSp53, and vinculin from actin filament images were similar to the truth images, indicating that the convolutional networks were able to predict the actin-related protein localization from actin filament images. However, the accuracy of translation was not at pixel resolution, which is thought to be the target of future studies. In contrast, the large filamentous structures of microtubules were not accurately predicted, which might imply indirect connections between actin filaments and microtubules. Collectively, this study suggests that image translation by convolutional networks can predict the localization of functionally related proteins, and the convolutional networks might be used to describe the relationships between the proteins by their localization.

## Results

### Prediction of WAVE2 Localization From Images of Actin Filaments

We used Swiss 3T3 cells because they form lamellipodia upon the activation of Rac1 (Ridley et al., 1992). We introduced a constitutively active Rac1 mutant into Swiss 3T3 cells to induce lamellipodia. After chemical fixation, the cells were stained with phalloidin and an anti-WAVE2 antibody to visualize actin filaments and WAVE2, respectively. The fan-shaped actin filament substructures at the cell periphery, which were assumed to be lamellipodia, had WAVE2 (Figures 1B,C). However, not all actin filaments have WAVE2.

For an initial test for the image translations from actin filament images to WAVE2 images, the pairs of images of actin filaments and WAVE2 were taken and used for the training of the pix2pix conditional GAN model. The detailed methodology is described in the Conditional GAN subsection of the “Materials and Methods” section (Hiasa et al., 2019). The translation performance was estimated by four-fold cross-validation, with 772 paired images of actin filaments and WAVE2. In each subset, the training set comprised 579 images, of which 15% were used as the validation set. No augmentation of the images of the Swiss 3T3 cells was performed. The remaining 193 images were used as the test set. The number of iterations, which corresponded to the epoch number for the training, was 200,000. This process was repeated four times for four-fold cross-validation. As the number of iterations increased, the similarity between the generated and actual WAVE2 images increased (Figure 1D). The generated WAVE2 final images were similar to those obtained using antibody staining.

**FIGURE 2:**
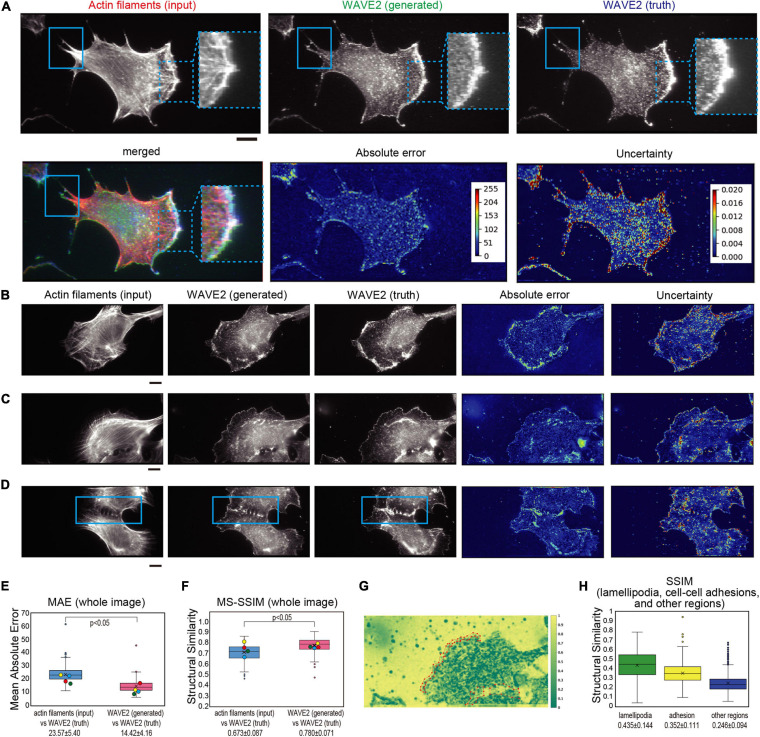
Generation of WAVE2 images from actin filaments in Swiss 3T3 cells. **(A)** Generation of a WAVE2 image by pix2pix from an actin filament image. The cells were stained with phalloidin for actin filaments and with an anti-WAVE2 antibody after fixation and permeabilization. An input image (actin filament image), an output image (generated WAVE2 image), a ground truth image (WAVE2 immunostained image), an absolute error image, and an uncertainty image are shown. The microspikes in the lamellipodia are marked with dashed squares, and the protrusions outside of the lamellipodia are marked with solid squares. Absolute error represents the difference in WAVE2 values in each pixel. Uncertainty in image generation represents the fluctuation of WAVE2 values based on various “dropouts” in convolutional neural networks, i.e., the robustness of the generation in each pixel. With higher values of absolute errors and uncertainty, the color of the heat map becomes closer to red. The merged image of actin filament, WAVE2 (generated), and WAVE2 (truth) was also shown to visualize the co-localization. Scale bar, 10 μm. **(B,C)** Generation of WAVE2 images of cells with various sizes of lamellipodia, as shown in panel **(A)**. Scale bars, 10 μm. **(D)** Generation of a WAVE2 image of cells that formed a cell-cell adhesion marked by a rectangle. Scale bar, 10 μm. **(E)** Box plot of the mean absolute error (MAE) between the generated and actual WAVE2 images, as well as between images of actin filaments (input) and actual WAVE2 as a reference. Quantification was performed for all images with four-fold cross-validation (*n* = 772). The mean values are shown at the bottom. **(F)** Box plot of the multi-scale structural similarity index measure (MS-SSIM) value between the generated and actual WAVE2 images, as well as between images of actin filaments and actual WAVE2 as a reference. Quantification was performed for all images with four-fold cross-validation (*n* = 772). The data points represent the MS-SSIM values for the generated images in panels **(A–D)**. The mean values are shown at the bottom. In panels **(E,F)**, the yellow, red, blue, and green circles indicate the values for images in panels **(A–D**), respectively. **(G)** Representative SSIM map corresponding to the image in panel **(C)**, showing the structural similarity at each 11 × 11 pixel window. Regions of lamellipodia are marked with polygons. **(H)** Box plot of the SSIM values from 1,926 pairs of lamellipodia, cell-cell adhesions, and other cellular regions. Statistical significance is shown by *p* < 0.05 by two-sample equal variance two-tailed Student’s *t*-test.

Figure 2 presents examples of the generated WAVE2 images, which also show the true actin filaments and true WAVE2 at lamellipodia, microspikes, cellular protrusions, and cell-cell adhesions. WAVE2 showed prominent localization at lamellipodia, and WAVE2 localization was clearly generated at the edge of the cells by the trained pix2pix model (Figures 2A–C). Regardless of the size of the lamellipodia, the pix2pix model predicted the localization of WAVE2 (Figures 2A–C). WAVE2 was not only localized in lamellipodia but also in other subcellular structures of actin filaments, including the tips of microspikes or filopodia within the lamellipodia (Figure 2A; Nakagawa et al., 2003; Nozumi et al., 2003). The dashed square in Figure 2A indicates that the pix2pix model could predict WAVE2 localization at the microspike structures in lamellipodia. Interestingly, the solid square in Figure 2A indicates that the protrusions outside of the lamellipodia were also predicted to have WAVE2 and indeed had real WAVE2. WAVE2 also reportedly functions at the cell-cell junctions (Yamazaki et al., 2007; Nishimura et al., 2016). The two cells were in contact with each other, with WAVE2 localization at the contact sites (Figure 2D). WAVE2 localization was clearly generated between the cell-cell contacts (Figure 2D). In each image, the overall predicted WAVE2 localization by pix2pix appeared to be quite similar to the real WAVE2 localization detected by antibody staining. Together, these facts suggested that pix2pix could predict the localization of WAVE2 not only in the lamellipodia but also in other cellular structures from actin filament images.

### Performance of the Prediction of WAVE2 Localization

We evaluated the prediction accuracy of each pixel. The absolute error in Figures 2A–D is the difference between the generated and true WAVE2 in each pixel, which at lamellipodia was higher than that of the background (Figures 2A–D). Another estimation of accuracy was based on the robustness of the prediction. The uncertainty of such translation was successfully estimated using Bayesian convolutional neural networks (Bayesian CNNs), based on the U-net architecture combined with the Monte Carlo dropout of the network layers (Hiasa et al., 2019). The dropout (removal) of the network layers results in different outputs; however, the high probability output contains less dependency on the alterations of the network layers, resulting in less uncertainty in the output. The uncertainty in the predictions of WAVE2 localization was also high in the lamellipodia (Figures 2A–D).

Despite the recognizable similarity, the errors were higher for the pixels of WAVE2 localization. These higher absolute errors and uncertainty at the pixels of WAVE2 localization compared to those at the background appeared to suggest that the intensity of WAVE2 localization was not predicted in the absolute values at a pixel resolution; instead, the prediction was more qualitative, reflecting the context of actin filaments for WAVE2 localization. Therefore, the absolute error would be caused by the aleatoric uncertainty from the randomness of the measurements rather than the epistemic uncertainty of the prediction.

Then, the overall image prediction was summarized by the mean absolute error (MAE) between the generated and truth WAVE2 images. MAE is the mean absolute difference in pixel values, which is related to the absolute errors in each image. Therefore, a smaller MAE indicates a higher similarity between the two images. We also employed another estimation, the structural similarity index measurement (SSIM). SSIM is based on the variance in the pixel values, and the multi-scale SSIM (MS-SSIM) uses SSIM of various scales, i.e., image resolution, to synthesize the similarity at various scales (Wang et al., 2003). A higher MS-SSIM indicates greater similarity in perceived quality. The MAE between the generated and truth images was statistically lower, and the MS-SSIM between the generated and truth images was statistically higher than those between the true actin filament (input) and true WAVE2 images, indicating that the pix2pix generated more similar images to the truth images than to the input images (Figures 2E,F). The MAE and MS-SSIM between WAVE2 images and random noise were significantly inferior to those between the generated and true WAVE2 images, indicating the validity of these estimations (Supplementary Figure 1).

During training, the MAE and MS-SSIM values were progressively improved by increasing the number of iterations (Supplementary Figure 2). At 0–25,000 iterations, the actin filamentous structures of the input images were still strongly reflected in the generated WAVE2 images (Figure 1D). These filamentous structures disappeared after 100,000 iterations (Figure 1D). Evaluations with MAE and MS-SSIM showed that they gradually improved as the iteration numbers increased, although no significant difference was observed between the MS-SSIM at 100,000 and 200,000 iterations (Supplementary Figure 2), suggesting that these values were not suitable for the evaluation of the recognizable image quality. Overall, these results suggested that pix2pix successfully produced WAVE2 images that were similar to true WAVE2 images compared to the input actin filament images, although the accuracy was not at pixel resolution.

Subsequently, we analyzed the performance of WAVE2 localization prediction at the subcellular level, which was the intermediate between the pixel and the whole image levels, as described above. The SSIM was calculated for each 11 × 11-pixel window to generate the SSIM map, and the representative analysis corresponding to Figure 2C is shown in Figure 2G. Then, the images of the ground truth were manually annotated using Labelme (Russell et al., 2008), which is a software used to assist in the extraction of the coordinates of the manually determined region of interest as polygons, saving the lamellipodia region information by the human eye (Figure 2G). The SSIM of these manually annotated lamellipodia and cell-cell adhesions was compared with the SSIM in the other cellular regions (Figure 2H). The average SSIMs of lamellipodia and cell-cell adhesion sites were higher than the average SSIMs in the non-lamellipodia regions (Figure 2H), suggesting that the GAN generated images based on meaningful localizations.

### Comparison With U-Net

Pix2pix has two components: a generator and a discriminator. The generator is similar to an original U-net (Ronneberger et al., 2015). To examine the contribution of the discriminator in GAN for image generation by pix2pix, we trained the model with a generator alone, i.e., only a U-net structure. The condition is the same as described above, except that the contribution of the discriminator to be none. The U-net-only model generated a blurry image compared to those by pix2pix (Supplementary Figure 3A). However, WAVE2 at the leading edge was predicted using the U-net-only model. Therefore, the U-net-only model was able to predict WAVE2 localization in lamellipodia. The difference between the U-net-only model and pix2pix appeared to be the dot-like localization of WAVE2 inside the cell, which was the blurred localization of WAVE2 in the U-net-only model. Importantly, the prediction of dot-like localization of WAVE2 was not accurate at pixel resolution, as described for Figure 2. In addition, the generated WAVE2 localization by the U-net-only model inside the cells was partially filamentous, reflecting the localization of actin filaments in the input images.

The images obtained using the U-net-only model and pix2pix were evaluated using MAE and MS-SSIM (Supplementary Figures 3B,C). The U-net only model showed higher performance than the pix2pix model in MS-SSIM values, which would be the result of the inaccurate prediction of the dot-like localization of WAVE2 by pix2pix. Therefore, to estimate the complexity of the generated image, we compared the entropy of the label image, the image generated by pix2pix, and the image generated by the U-net-only model (Supplementary Figure 3D). The entropy showed that the generated image of the pix2pix model was closer to the label image than the generated image of the U-net-only model. Therefore, we thought that the U-net-only model did not express the complexity of the original WAVE2 stained image, but pix2pox did not generate accurate WAVE2 localization at pixel resolution.

### Application to the Localization of Actin Filaments by Lifeact and to That of IRSp53

**FIGURE 3:**
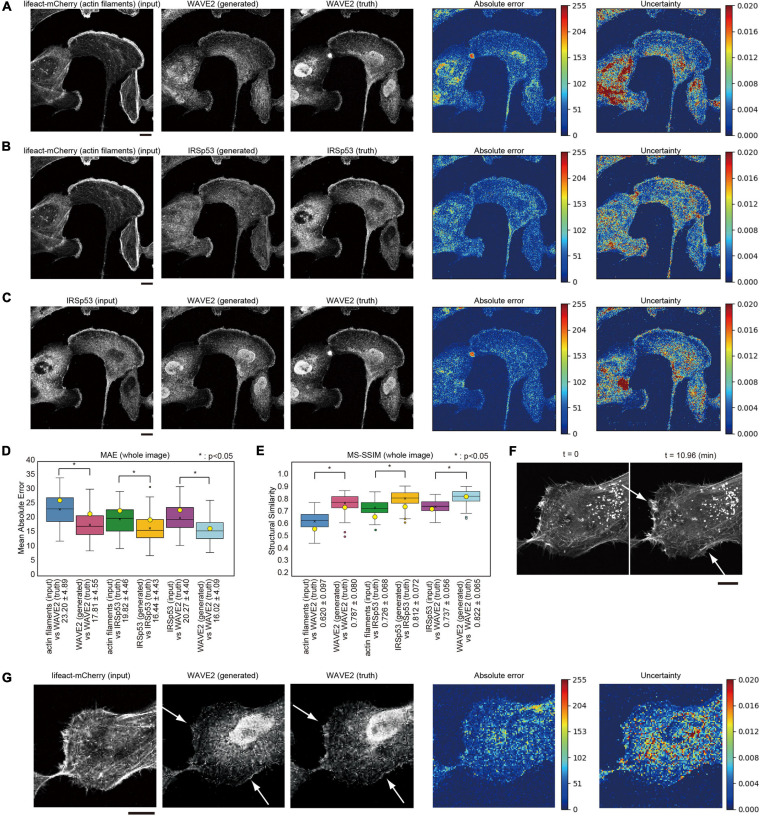
Generation of WAVE2 and IRSp53 images from actin filaments and WAVE2 images in IRSp53-expressing U251 cells. **(A)** Generation of a WAVE2 image from an actin filament image that was visualized by lifeact-mCherry. The IRSp53-knocked-out U251 cells re-expressing GFP-tagged IRSp53 and lifeact-mCherry were stained with an anti-WAVE2 antibody after fixation and permeabilization. The results are depicted as in Figure 2A. Scale bar, 10 μm. **(B)** Generation of an IRSp53 image from an actin filament-stained image. Scale bar, 10 μm. **(C)** Image generation into a WAVE2 image from an IRSp53-stained image. Scale bar, 10 μm. **(D)** Box plot of the MAE of the entire images in four-fold cross-validation (*n* = 100). **(E)** Box plot of the MS-SSIM of the entire images in four-fold cross-validation (*n* = 100). The data points represent the MS-SSIM values for the generated images in panels **(A–C)**. In panels **(D,E)**, the yellow circles indicate the values for images in panels **(A–C)**. Statistical significance is shown by *p* < 0.05 (*) by two-sample equal variance two-tailed Student’s *t*-test. **(F)** Lamellipodia structures (arrows) observed in the live imaging of lifeact-mCherry. Scale bar, 10 μm. **(G)** Generation of a WAVE2 image from a lifeact-mCherry image. The cells observed in panel **(F)** were fixed, permeabilized, and immunostained for WAVE2 (truth). The arrows indicate lamellipodia. Scale bar, 10 μm.

To examine the generalization of this method, we trained the pix2pix model using the images of glioma U251 cells for another regulator of WAVE2, IRSp53 (Miki et al., 2000; Suetsugu et al., 2006). IRSp53-knockout U251 cells were prepared, and IRSp53 expression was restored by stable expression of GFP-IRSp53. The cells were then further stably labeled with a lifeact tagged with mCherry (lifeact-mCherry) to visualize actin filaments (Riedl et al., 2008). These U251 cells were cultured in serum and fixed for the observation of lamellipodia without forced activation of Rac1. WAVE2 localization was identified using antibody staining. The images of the actin filaments by lifeact, IRSp53 by GFP, and WAVE2 by antibody staining were similar in lamellipodia but not identical in the other regions of the cells. These images were subjected to the machine learning for image translation using pix2pix. We attempted to translate the images of actin filaments (lifeact) to WAVE2, actin filaments to IRSp53, and IRSp53 to WAVE2. Translation performance was estimated using four-fold cross-validation. In total, 100 images were obtained, and 75 were subjected to training. The 75 images were augmented seven-fold by rotations at 90° steps and vertical and horizontal flipping, resulting in a training dataset composed of 525 images, of which 15% were used as the validation set. The remaining 25 images were used as the test set. The results showed that a lifeact image could produce images of WAVE2 and IRSp53 (Figures 3A,B). Furthermore, pix2pix translated the IRSp53 images into WAVE2 images (Figure 3C). WAVE2 and IRSp53 were well co-localized at lamellipodia but were not well co-localized in the other regions of the cells. The prediction of lamellipodia localization of these proteins, as well as the localization at the cytosol, was regarded as having good quality, because the MAE values between the truth and generated images were lower than the MAE values between the input and truth images (Figure 3D), and because the MS-SSIM values between the generated and truth images were higher than those between the input and truth images (Figure 3E). However, the staining around the nucleus appeared to be predicted with less accuracy than that at the cell periphery (Figures 3A,B), which might indicate the lesser relationships of these proteins in the nucleus.

We observed the lifeact-mCherry in live cells to identify lamellipodia at the leading edge (Figure 3F). The cells were then fixed, permeabilized, and stained for WAVE2 localization. Permeabilization slightly altered the lifeact images because the free lifeact in the cytosol was probably removed by permeabilization. The active lamellipodia region was stained with WAVE2, and the actin filament images for these lamellipodia were able to generate the WAVE2 image by using the trained model as described above (Figure 3G). From these results, we concluded that pix2pix could specifically predict WAVE2 localization at the leading edge of lamellipodia under different conditions.

### Application to Vinculin and Tubulin Localizations

**FIGURE 4:**
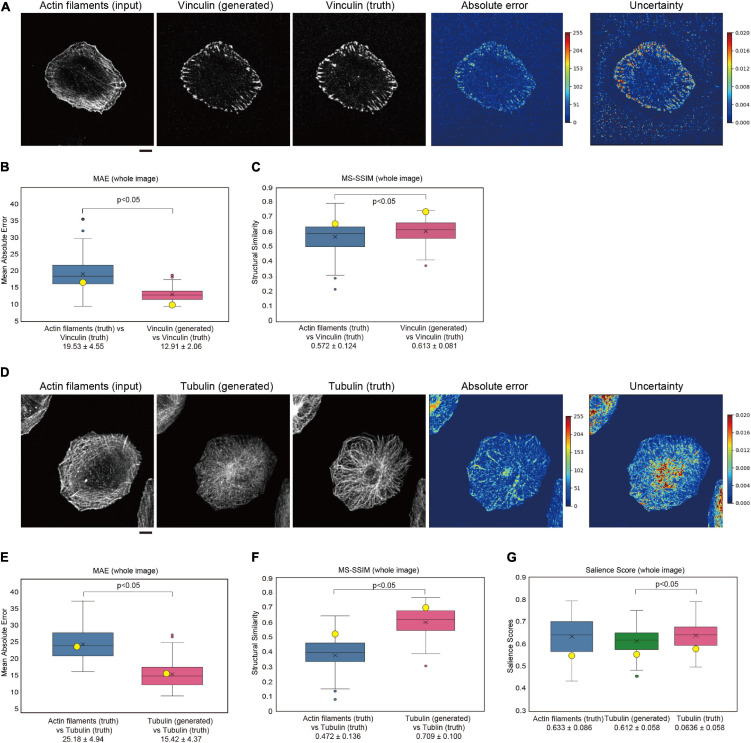
Generation of vinculin and tubulin images in U251 cells. **(A)** Generation of a vinculin image from an actin filament-stained image. U251 cells were stained with phalloidin for actin filaments and with an anti-vinculin antibody after fixation and permeabilization. The results are depicted as in Figure 2A. Scale bar, 10 μm. **(B)** Box plot of the MAE of entire images in four-fold cross-validation for panel **(A)** (*n* = 100). **(C)** Box plot of the SSIM of entire images in four-fold cross-validation for panel **(A)** (*n* = 100). The data point represents the MS-SSIM values for the generated images in panel **(A)**. **(D)** Generation of a tubulin image from an actin filament-stained image. U251 cells were stained with phalloidin for actin filaments and with an anti-α-tubulin antibody after fixation and permeabilization. The results are depicted as in Figure 2A. Scale bar, 10 μm. **(E)** Box plot of the MAE of entire images in four-fold cross-validation for panel **(D)** (*n* = 100). **(F)** Box plot of the SSIM of entire images in four-fold cross-validation for panel **(D)** (*n* = 100). The data point represents the MS-SSIM values for the generated images in panel **(D)**. **(G)** Box plot of the salience score of entire images in four-fold cross-validation for panel **(D)** (*n* = 100). In panels **(B,C,E–G)**, the yellow circles indicate the values for images in panels **(A,D)**. Statistical significance is shown by *p* < 0.05 by two-sample equal variance two-tailed Student’s *t*-test.

Focal adhesions, which contain vinculin protein, are known to promote lamellipodia formation. To examine whether GAN could be applied to other molecules that are related to lamellipodia, we trained the model between actin filaments and vinculin staining. The translation performance of the model, trained by 100-paired actin filament and vinculin images of U251 cells, was estimated by four-fold cross-validation as for the IRSp53 and WAVE2 analysis. Pix2pix succeeded in generating vinculin images from the actin filament images (Figure 4A). The prediction of vinculin was regarded as having good quality, as judged by the MAE and MS-SSIM values (Figures 4B,C), as well as by human eye recognition.

To examine whether GAN could be applied to other molecules that are not strongly related to actin filaments, we trained the model between actin filaments and tubulin staining of U251 cells as was performed for the IRSp53, WAVE2, and vinculin analyses. The trained pix2pix model generated a tubulin-like image from the actin filament images (Figures 4D–F). However, the generated images did not reflect the features of filamentous and radial tubulin distribution (Figures 4D–F). On the other hand, the MAE and MS-SSIM values indicated a good quality of image translation, and the generated tubulin images were apparently closer to the tubulin images than the actin images. Therefore, the MAE and MS-SSIM might not reflect cell-wide features, such as filamentous radial localization. Then, we tested the salience score, showing the local symmetry of the images (Rezanejad et al., 2019; Wilder et al., 2019). The generated tubulin images had a smaller score than the truth tubulin images (Figure 4G). However, further development of the index for evaluating the similarity between images would be required in the future.

## Discussion

In this study, we predicted the subcellular localization of WAVE2, IRSp53, and vinculin, which were established regulators of lamellipodia, using convolutional networks. The generated images had striking similarities to the truth images, although, at this moment, the generated images did not have accuracy at pixel resolution, which would be in future development. Therefore, the prediction of these localizations by image translation was suggested to be used for global estimation of protein localization, which would include an annotation of lamellipodia by protein localization among actin cytoskeletal structures. Experts in the field will easily distinguish lamellipodial actin filaments from non-lamellipodial ones, but sometimes lamellipodia are not obvious to the untrained eyes. Furthermore, this method can be used to label lamellipodia in live cells to quantify the degree of lamellipodia formation if the computation speed is sufficient.

The prediction of WAVE2 localization was independent of lamellipodia size (Figure 2). This independence could be related to the kernel size required for the computation. The kernel size of the algorithm, which used the four-pixel window, was equivalent to ∼1.9 μm^2^. The features of actin filaments in lamellipodia, i.e., branched filaments, were thought to be within this window. Thus, various sizes of lamellipodia could be predicted for WAVE2 localization. Vinculin and IRSp53 localizations were also thought to be predictable with such features of actin filaments in a four-pixel window.

Thus, the continuous features above this size are supposed to be difficult to be predicted. We attempted to predict microtubule localization from actin filament localization; however, the predicted localization of microtubules was not filamentous. These non-continuous filaments might arise from the kernel size. Alternatively, microtubules were not directly linked to actin filaments, in contrast to the regulators of actin filaments such as WAVE2, IRSp53, and vinculin, resulting in inaccurate prediction because of the potential shortage of information related to microtubules. Nevertheless, it should be noted that the image comparison statistics, MAE and MS-SSIM, suggested that the generated tubulin images were more similar to the truth tubulin images than to the actin filament images.

If the difference between the experimental and predicted images resulted from the mutually independent localization and function, then the convolutional network might be used as a tool to discover a localization and a function that could be independent of each other. The possible failure of the prediction of the nuclear staining of WAVE2 in U251 cells might indeed suggest the actin filament-independent function of WAVE2 (Figure 3), as the nuclear localization of WAVE1 has been reported (Miyamoto and Gurdon, 2013). However, the nuclear staining of WAVE2 might represent non-specific staining, which was also thought to be independent of actin filaments. Such nuclear staining of WAVE2 was not observed in Swiss 3T3 cells (Figure 2), which might imply both that nuclear WAVE2 in U251 cells could be a result of non-specific staining and that the nuclear function of WAVE2 might differ between U251 and Swiss 3T3 cells. In both cases, the prediction was thought to require training depending on the cell types and might reflect the specific observation of the cells. In addition, these inconsistencies in WAVE2 localization between the generated and truth images might be due to insufficient learning and randomness in experimental errors. Therefore, further investigations, especially the development of statistics that could evaluate such image features, would be required to explore further the idea that the difference between the predicted and truth images results from the functional independence of the observed pair of molecules.

With the development of statistics for image comparison, as well as the refinement of the prediction into pixel resolutions as well as into cell-wide features that were seen in tubulin images, the prediction of protein localization could have great potential for understanding the relationships between proteins and molecules. Furthermore, the prediction of molecule localization was also considered as artificial staining of cells. Labeling with antibodies was normally limited to several proteins. In contrast, artificial staining could predict an unlimited number of protein localizations from single staining, which would be useful for detecting the relationships between many molecules after future development.

## Materials and Methods

### Cell Culture

Plat-E, Swiss 3T3, and U251 cells were cultured in Dulbecco’s modified Eagle’s medium (DMEM) (Nacalai Tesque, 08459-64), supplemented with 10% fetal calf serum (FCS) and penicillin-streptomycin (PS) (DMEM-10% FCS/PS) at 37°C in a 5% CO_2_ incubator. Plat-E, Swiss 3T3, and A549 cells were passaged every 4, 3, and 2 days, respectively.

### Retrovirus-Mediated Gene Transfer

Swiss 3T3 cells were transfected with the pMX-Myc-Rac1-CA vector (Suetsugu et al., 1999a). First, Plat-E cells were cultured overnight in a 12-well plate in DMEM containing 10% FCS/PS. For transfection, 100 μL of Opti-MEM with 1.6 μg of vector and 100 μL of Opti-MEM with 1 μL of 293 fectin transfection reagents (Thermo Fisher) were mixed, allowed to form a complex at room temperature for 20 min, and then added to the Plat-E cells in 0.8 ml medium (Kitamura et al., 2003). After 48 h, the culture supernatant was filtered using a 0.22 μm filter and added to the cells in 1.2 ml medium with polybrene at a concentration of 8 μg/mL. After 24 h, the medium was replaced with fresh DMEM containing 10% FCS/PS. After an additional 24 h, the cells were replated on a 24-well plate containing a coverslip (Matsunami) and cultured for another 48 h.

The IRSp53-knockout U251 cell line expressing GFP-IRSp53 was established using CRISPR/Cas9-mediated genome editing. The guide RNA targeting the second exon (29th amino-acid residue) of IRSp53 (CCATGGCGATGAAGTTCCGG) was designed using the server http://crispr.mit.edu (Hsu et al., 2013) and inserted into the pX330 vector, which was transfected into the cells and then cloned (Mashiko et al., 2013). The expression of GFP-IRSp53 and lifeact-mCherry was performed using the retrovirus as described above, and then clones were isolated using a fluorescence-activated cell sorter.

### Immunofluorescent Staining of Swiss 3T3 and U251 Cells

The cells were fixed with 4% paraformaldehyde in PBS for 20 min at room temperature. Subsequently, the cells were permeabilized with 0.5% Triton X-100 in PBS for 20 min at room temperature with gentle shaking. Then, the cells were washed with 0.1% Triton X-100 in PBS (PBS-T). Next, PBS containing 3% bovine serum albumin and 10% goat serum was added to block the cells for 1 h with gentle shaking. The cells were then washed with PBS-T. The primary antibody, rabbit anti-WAVE2 antibody (Cell Signaling, # 3659S), mouse anti-vinculin (SIGMA, V 9131), and mouse anti-alpha-tubulin (SIGMA, clone DM1A) was diluted 100-, 200-, and 500-fold, respectively, in the blocking solution, incubated for 1 h with gentle shaking, and then washed three times with PBS-T. The secondary antibody, Alexa Fluor 488-goat anti-rabbit or mouse IgG antibody (highly cross-absorbed, Thermo Fisher) diluted 400-fold, and rhodamine-phalloidin (Thermo Fisher) for actin filament detection, diluted 1,000-fold in the blocking solution, were added and then incubated for 1 h with gentle shaking in the dark. The cells were then washed with PBS-T and mounted on a glass slide, using Prolong Diamond Antifade Mountant with DAPI (Thermo Fisher), allowed to solidify at room temperature overnight, and then stored at 4°C. Swiss 3T3 cells were observed using an IX81 fluorescence microscope (OLYMPUS) with W-View Gemini (Hamamatsu Photonics). U251 cells were observed using an FV1000 confocal microscope (Olympus).

### Conditional GANs

The purpose of this study was to determine the conditional distribution of WAVE2 based on actin filaments. The pix2pix conditional GAN (Isola et al., 2017) allows the patch discriminator to capture the Markov property of the image as an adversarial loss, allowing the transformed image to maintain high spatial frequencies. The formula for this adversarial learning is as follows:

(1) $$\hat{G}=\arg\min_{G}\max_{D}{ℒ}_{GAN}\left(G,D\right)+\lambda {ℒ}_{L1}\left(G\right),$$

where a generator *G* translates images of actin filaments *x* to WAVE2 images *y*, which are trained to translate images of actin filaments that a discriminator *D* cannot distinguish from the “real” WAVE2 images by antibody staining, as follows:

(2) $${ℒ}_{GAN}\left(G,D\right)={𝔼}_{x,y∼p_{data}\left(x,y\right)}\left[\log D\left(x,y\right)\right]+{𝔼}_{x∼p_{data}\left(x\right),z∼p_{z}\left(z\right)}[\log\left(1-D\left(x,G\left(x,z\right)\right)\right].$$

In addition to the adversarial loss, the conditional loss, which is the similarity between the “fake” and “real” WAVE2 images, is introduced as follows:

(3) $${ℒ}_{L1}\left(G\right)={𝔼}_{x,y∼p_{data}\left(x,y\right),z∼p_{z}\left(z\right)}\left[{||y-G\left(x,z\right)||}_{1}\right],$$

where *z* denotes the random noise.

We primarily followed this framework and extended the generator and discriminator networks. Here, the generator was replaced with a Bayesian U-Net (Hiasa et al., 2019) for the uncertainty estimation. Spectral normalization (Miyato et al., 2018) was applied to the patch discriminator to stabilize the optimization. In the inference phase, the predictive distribution is expressed as

(4) $$\mu \left[y\right]={𝔼}_{z∼p_{z}}G\left(x,z\right),var\left[y\right]={𝔼}_{z∼p_{z}}\left(G\left(x,z\right)-\hat{y}\right),$$

where μ and *var* denote the mean and variance, respectively.

WAVE2 and actin filament images were downscaled to 256 × 256 pixels and normalized such that the intensities of the 1st and 99th percentiles were mapped to [−1, 1]. Data augmentation was applied based on spatial transforms, including the translation of [−10, +10]% of the image size, rotation of [−10, +10]°, scale of [−10, +10]%, shear transformation with a shear angle of [−π/16, +π/16] rad, and flipping in the horizontal and vertical directions. The kernel size was 4 pixels, that is, ∼1.9 μm^2^. The codes that were used, including the details of each network and training manner, are available at https://github.com/yuta-hi/bayesian_unet.

### Estimation of Errors

The results were evaluated based on the MAE and SSIM. The MAE shows the absolute error in the brightness value of each pixel and is expressed as

(5) $$\text{MAE}\left(f_{i},y_{i}\right)=\frac{1}{n}\sum_{k-1}^{n}\left|f_{i}-y_{i}\right|,$$

where *f*_*i*_ and *y*_*i*_ denote the true and predicted values, respectively.

The SSIM indicates the similarity of the average, variance, and covariance of the surrounding pixels in terms of brightness, contrast, and structure. Thus, it is an index that incorporates the correlation not only with individual pixels but also with the surrounding pixels. The SSIM is expressed as

(6) $$\text{SSIM}\left(\text{x},\text{y}\right)=\frac{\left(2\mu_{x}\mu_{y}+C_{1}\right)\left(2\sigma_{xy}+C_{2}\right)}{\left(\mu_{x}^{2}+\mu_{y}^{2}+C_{1}\right)\left(\sigma_{x}^{2}+\sigma_{y}^{2}+C_{2}\right)},$$

where x and y are the ground truth (WAVE2) and predicted images, respectively, μ is the average pixel value, σ is the standard deviation of the pixel value, σ_*xy*_ is the covariance between x and y, C1 = (0.01 × L2), C2 = (0.03 × L2), and L is the dynamic range of the images (Wang et al., 2004). We used 8-bit images; hence, *L* = 255.

Multi-scale SSIM was calculated by the SSIM at five scales, which were down sampling of images by a factor of two with each scaling.

(7) $$\text{MS-}\,\text{SSIM}\,\left(\text{x},\text{y}\right)={\left[l_{M}\,\left(x,y\right)\right]}^{\alpha_{\text{M}}}\,\prod_{J=1}^{M}{\left[c_{J}\,\left(x,y\right)\right]}^{\beta_{j}}\,{\left[s_{j}\,\left(x,y\right)\right]}^{\gamma_{j}},$$

where M is the number of down sampling, α, β, and γ are equal values in each scale, and β_1_ = γ_1_ = 0.0448, β_2_ = γ_2_ = 0.2856, β_3_ = γ_3_ = 0.3001, β_4_ = γ_4_ = 0.2363, α_5_ = β_5_ = γ_5_ = 01333, which is derived from the Gaussian distribution with the assumption that the medium resolution is suitable for recognition (Wang et al., 2003).

Random noise images were obtained from the label image by random shuffling of each pixel.

The lamellipodia regions were manually annotated for SSIM calculations using Labelme (Russell et al., 2008)^1^ to extract the SSIM values at the lamellipodia.

Entropy represents the complexity of images. The entropy is expressed as

(8) $$H=-\sum p_{i}log_{2}p_{i},$$

where p_*i*_ is the probability of appearance of a particular pixel value, which is introduced as follows:

(9) $${\text{p}}_{\text{i}}=\frac{{\text{N}}_{\text{i}}}{\text{N}},$$

where N is the number of total pixels, and N_*i*_ is the number of particular pixel values.

The salience score was calculated using the local symmetry of the images (Rezanejad et al., 2019; Wilder et al., 2019). The contour for the salience score was generated by the banalization of the images, with a threshold level of 0.25, because the average threshold for binarization by the Otsu method was approximately 0.25.

Statistical significance is shown by *p* < 0.05 by two-sample equal variance two-tailed Student’s *t*-test.

## Data Availability Statement

The raw data supporting the conclusions of this article will be made available by the authors, without undue reservation.

## Author Contributions

KS, SJ, and TN performed the experiments. KS, YH, YO, SJ, and MS performed the computations. YS and SS supervised the project. All authors analyzed the results, created the figures, and wrote the manuscript.

## Conflict of Interest

The authors declare that the research was conducted in the absence of any commercial or financial relationships that could be construed as a potential conflict of interest.

## Publisher’s Note

All claims expressed in this article are solely those of the authors and do not necessarily represent those of their affiliated organizations, or those of the publisher, the editors and the reviewers. Any product that may be evaluated in this article, or claim that may be made by its manufacturer, is not guaranteed or endorsed by the publisher.

## Footnotes

1
https://github.com/wkentaro/Labelme

## Supplementary Material

Supplementary Figure 1 The MAE and MS-SSIM to random noise. **(A)** True and the generated WAVE2 images as in Figure 2 and the random noise image that was generated by shuffling the true WAVE2 image. **(B)** Box plot of the MAE of entire images for panel **(A)** (*n* = 772). **(C)** Box plot of the MS-SSIM of entire images for panel **(A)** (*n* = 772).

Supplementary Figure 2 The progress of image generation per iterations. **(A)** Box plot of the MAE of entire images in four-fold cross-validation for Figure 1D (*n* = 772). **(B)** Box plot of the MS-SSIM of entire images in four-fold cross-validation for Figure 1D (*n* = 772). Statistical significance is shown by *p* < 0.05 (*) by two-sample equal variance two-tailed Student’s *t*-test.

Supplementary Figure 3 The comparison between pix2pix and U-net. **(A)** Generation of a WAVE2 image by pix2pix model and U-net only model from an actin filament image. An input image (actin filament image), a ground truth image (WAVE2 immunostained image), a pix2pix output image, and a U-net only model output image. Scale bar, 10 μm. **(B)** Box plot of the MAE of entire images in four-fold cross-validation for panel **(A)** (*n* = 772). **(C)** Box plot of the MS-SSIM of entire images in four-fold cross-validation for panel **(A)** (*n* = 772). **(D)** Box plot of the entropy of entire images in four-fold cross-validation for panel **(A)** (*n* = 772). Statistical significance is shown by *p* < 0.05 by two-sample equal variance two-tailed Student’s *t*-test.

## References

[B1] Bear J. E. Rawls J. F. Saxe C. L. III (1998). SCAR, a WASP-related protein, isolated as a suppressor of receptor defects in late Dictyostelium development. *J. Cell Biol.* 142 1325–1335. 10.1083/jcb.142.5.1325 9732292 PMC2149354

[B2] Brent R. Boucheron L. (2018). Deep learning to predict microscope images. *Nat. Methods.* 15 868–870. 10.1038/s41592-018-0194-9 30377365 PMC6322918

[B3] Camacho D. M. Collins K. M. Powers R. K. Costello J. C. Collins J. J. (2018). Next-generation machine learning for biological networks. *Cell* 173 1581–1592. 10.1016/j.cell.2018.05.015 29887378

[B4] Chen Z. Borek D. Padrick S. B. Gomez T. S. Metlagel Z. Ismail A. M. (2010). Structure and control of the actin regulatory WAVE complex. *Nature* 468 533–538. 10.1038/nature09623 21107423 PMC3085272

[B5] Christiansen E. M. Yang S. J. Ando D. M. Javaherian A. Skibinski G. Lipnick S. (2018). In silico labeling: predicting fluorescent labels in unlabeled images. *Cell* 173 792–803.e19. 10.1016/j.cell.2018.03.040 29656897 PMC6309178

[B6] Goodfellow I. Pouget-Abadie J. Mirza M. Xu B. Warde-Farley D. Ozair S., et al. (eds) (2014). “Generative adversarial nets,” in *Proceedings of the 27th International Conference on Neural Information Processing Systems*, Montreal, QC.

[B7] Gunning P. W. Ghoshdastider U. Whitaker S. Popp D. Robinson R. C. (2015). The evolution of compositionally and functionally distinct actin filaments. *J. Cell Sci.* 128 2009–2019. 10.1242/jcs.165563 25788699

[B8] Hall A. (1998). Rho GTPase and the actin cytoskeleton. *Science* 279 509–514. 9438836 10.1126/science.279.5350.509

[B9] Hiasa Y. Otake Y. Takao M. Ogawa T. Sugano N. Sato Y. (2019). “Automated muscle segmentation from clinical CT using bayesian U-net for personalized musculoskeletal modeling,” in *IEEE Transactions on Medical Imaging*, 39, 1030–1040. 10.1109/TMI.2019.2940555 31514128

[B10] Hsu P. D. Scott D. A. Weinstein J. A. Ran F. A. Konermann S. Agarwala V. (2013). DNA targeting specificity of RNA-guided Cas9 nucleases. *Nat. Biotechnol.* 31 827–832. 10.1038/nbt.2647 23873081 PMC3969858

[B11] Innocenti M. Zucconi A. Disanza A. Frittoli E. Areces L. B. Steffen A. (2004). Abi1 is essential for the formation and activation of a WAVE2 signalling complex. *Nat. Cell Biol.* 6 319–327. 15048123 10.1038/ncb1105

[B12] Ismail A. M. Padrick S. B. Chen B. Umetani J. Rosen M. K. (2009). The WAVE regulatory complex is inhibited. *Nat. Struct. Mol. Biol.* 16 561–563. 10.1038/nsmb.1587 19363480 PMC2716658

[B13] Isola P. Zhu J.-Y. Zhou T. Efros A. A. (eds) (2017). “Image-to-image translation with conditional adversarial networks,” in *Proceedings of the IEEE Conference on Computer Vision and Pattern Recognition*, (Honolulu, HI: IEEE).

[B14] Kitamura T. Koshino Y. Shibata F. Oki T. Nakajima H. Nosaka T. (2003). Retrovirus-mediated gene transfer and expression cloning: powerful tools in functional genomics. *Exp. Hematol.* 31 1007–1014. 14585362

[B15] Kobayashi H. Lei C. Wu Y. Mao A. Jiang Y. Guo B. (2017). Label-free detection of cellular drug responses by high-throughput bright-field imaging and machine learning. *Sci. Rep.* 7:12454. 10.1038/s41598-017-12378-4 28963483 PMC5622112

[B16] Machesky L. M. Insall R. H. (1998). Scar1 and the related Wiskott-Aldrich syndrome protein, WASP, regulate the actin cytoskeleton through the Arp2/3 complex. *Curr. Biol.* 8 1347–1356. 10.1016/s0960-9822(98)00015-3 9889097

[B17] Mashiko D. Fujihara Y. Satouh Y. Miyata H. Isotani A. Ikawa M. (2013). Generation of mutant mice by pronuclear injection of circular plasmid expressing Cas9 and single guided RNA. *Sci. Rep.* 3:3355. 10.1038/srep03355 24284873 PMC3842082

[B18] Miki H. Suetsugu S. Takenawa T. (1998). WAVE, a novel WASP-family protein involved in actin reorganization induced by Rac. *EMBO J.* 17 6932–6941. 10.1093/emboj/17.23.6932 9843499 PMC1171041

[B19] Miki H. Yamaguchi H. Suetsugu S. Takenawa T. (2000). IRSp53 is an essential intermediate between Rac and WAVE in the regulation of membrane ruffling. *Nature* 408 732–735. 10.1038/35047107 11130076

[B20] Miyamoto K. Gurdon J. B. (2013). Transcriptional regulation and nuclear reprogramming: roles of nuclear actin and actin-binding proteins. *Cell. Mol. Life Sci.* 70 3289–3302. 10.1007/s00018-012-1235-7 23275942 PMC3753470

[B21] Miyato T. Kataoka T. Koyama M. Yoshida Y. (2018). Spectral normalization for generative adversarial networks. *arXiv* [Preprint] arXiv:180205957,

[B22] Moen E. Bannon D. Kudo T. Graf W. Covert M. Van Valen D. (2019). Deep learning for cellular image analysis. *Nat. Methods* 16 1233–1246. 10.1038/s41592-019-0403-1 31133758 PMC8759575

[B23] Nakagawa H. Miki H. Nozumi M. Takenawa T. Miyamoto S. Wehland J. (2003). IRSp53 is colocalised with WAVE2 at the tips of protruding lamellipodia and filopodia independently of Mena. *J. Cell Sci.* 116(Pt 12) 2577–2583. 10.1242/jcs.00462 12734400

[B24] Nishimoto S. Tokuoka Y. Yamada T. G. Hiroi N. F. Funahashi A. (2019). Predicting the future direction of cell movement with convolutional neural networks. *PLoS One* 14:e0221245. 10.1371/journal.pone.0221245 31483827 PMC6726366

[B25] Nishimura T. Ito S. Saito H. Hiver S. Shigetomi K. Ikenouchi J. (2016). DAAM1 stabilizes epithelial junctions by restraining WAVE complex-dependent lateral membrane motility. *J. Cell Biol.* 215 559–573. 10.1083/jcb.201603107 27807130 PMC5119936

[B26] Nozumi M. Nakagawa H. Miki H. Takenawa T. Miyamoto S. (2003). Differential localization of WAVE isoforms in filopodia and lamellipodia of the neuronal growth cone. *J. Cell Sci.* 116(Pt 2) 239–246. 10.1242/jcs.00233 12482910

[B27] Pärnamaa T. Parts L. (2017). Accurate classification of protein subcellular localization from high-throughput microscopy images using deep learning. *G3 (Bethesda)* 7 1385–1392. 10.1534/g3.116.033654 28391243 PMC5427497

[B28] Pollard T. D. Borisy G. G. (2003). Cellular motility driven by assembly and disassembly of actin filaments. *Cell* 112 453–465. 12600310 10.1016/s0092-8674(03)00120-x

[B29] Rezanejad M. Downs G. Wilder J. Walther D. B. Jepson A. Dickinson S. (eds) (2019). “Scene categorization from contours: medial axis based salience measures,” in *Proceedings of the 2019 IEEE/CVF Conference on Computer Vision and Pattern Recognition (CVPR)*, (Long Beach, CA: IEEE).

[B30] Ridley A. J. (2011). Life at the leading edge. *Cell* 145 1012–1022. 10.1016/j.cell.2011.06.010 21703446

[B31] Ridley A. J. Paterson H. F. Johnston C. L. Diekmann D. Hall A. (1992). The small GTP-binding protein rac regulates growth factor-induced membrane ruffling. *Cell* 70 401–410. 10.1016/0092-8674(92)90164-8 1643658

[B32] Riedl J. Crevenna A. H. Kessenbrock K. Yu J. H. Neukirchen D. Bista M. (2008). Lifeact: a versatile marker to visualize F-actin. *Nat. Methods* 5 605–607. 10.1038/nmeth.1220 18536722 PMC2814344

[B33] Ronneberger O. Fischer P. Brox T. (eds) (2015). “U-net: convolutional networks for biomedical image segmentation,” in *Proceedings of the International Conference on Medical Image computing and Computer-Assisted Intervention*, (Munich: Springer).

[B34] Russell B. C. Torralba A. Murphy K. P. Freeman W. T. (2008). LabelMe: a database and web-based tool for image annotation. *Int. J. Comput. Vis.* 77 157–173.

[B35] Sagawa Y. Hagiwara M. (2018). Face image generation system using attributes information with DCGANs. *Trans. Jpn. Soc. Kansei Eng.* 17 337–345.

[B36] Suetsugu S. Kurisu S. Oikawa T. Yamazaki D. Oda A. Takenawa T. (2006). Optimization of WAVE2 complex-induced actin polymerization by membrane-bound IRSp53, PIP(3), and Rac. *J. Cell Biol.* 173 571–585. 10.1083/jcb.200509067 16702231 PMC2063866

[B37] Suetsugu S. Miki H. Takenawa T. (1999a). Distinct roles of profilin in cell morphological changes: microspikes, membrane ruffles, stress fibers, and cytokinesis. *FEBS Lett.* 457 470–474. 10.1016/s0014-5793(99)01086-8 10471831

[B38] Suetsugu S. Miki H. Takenawa T. (1999b). Identification of two human WAVE/SCAR homologues as general actin regulatory molecules which associate with Arp2/3 complex. *Biochem. Biophys. Res. Commun.* 260 296–302. 10381382 10.1006/bbrc.1999.0894

[B39] Suetsugu S. Yamazaki D. Kurisu S. Takenawa T. (2003). Differential roles of WAVE1 and WAVE2 in dorsal and peripheral ruffle formation for fibroblast cell migration. *Dev. Cell* 5 595–609. 14536061 10.1016/s1534-5807(03)00297-1

[B40] Takai Y. Sasaki T. Matozaki T. (2001). Small GTP-binding proteins. *Physiol. Rev.* 81 153–208. 10.1152/physrev.2001.81.1.153 11152757

[B41] Takenawa T. Suetsugu S. (2007). The WASP-WAVE protein network: connecting the membrane to the cytoskeleton. *Nat. Rev. Mol. Cell Biol.* 8 37–48. 10.1038/nrm2069 17183359

[B42] Toratani M. Konno M. Asai A. Koseki J. Kawamoto K. Tamari K. (2018). A convolutional neural network uses microscopic images to differentiate between mouse and human cell lines and their radioresistant clones. *Cancer Res.* 78 6703–6707. 10.1158/0008-5472.Can-18-0653 30254144

[B43] Tsuda H. Hotta K. (eds) (2019). “Cell image segmentation by integrating pix2pixs for each class,” in *Proceedings of the IEEE Conference on Computer Vision and Pattern Recognition Workshops*, (Long Beach, CA).

[B44] Wang Z. Bovik A. C. Sheikh H. R. Simoncelli E. P. (2004). Image quality assessment: from error visibility to structural similarity. *IEEE Trans. Image Process.* 13 600–612. 15376593 10.1109/tip.2003.819861

[B45] Wang Z. Simoncelli E. P. Bovik A. C. (eds) (2003). “Multiscale structural similarity for image quality assessment,” in *Proceedings of the 37th Asilomar Conference on Signals, Systems & Computers*, Vol. 2003 (Pacific Grove, CA: IEEE), 9–12.

[B46] Wilder J. Rezanejad M. Dickinson S. Siddiqi K. Jepson A. Walther D. B. (2019). Local contour symmetry facilitates scene categorization. *Cognition* 182 307–317. 10.1016/j.cognition.2018.09.014 30415132

[B47] Yamazaki D. Oikawa T. Takenawa T. (2007). Rac-WAVE-mediated actin reorganization is required for organization and maintenance of cell-cell adhesion. *J. Cell Sci.* 120(Pt 1) 86–100. 10.1242/jcs.03311 17164293

[B48] Ziegler W. H. Liddington R. C. Critchley D. R. (2006). The structure and regulation of vinculin. *Trends Cell Biol.* 16 453–460. 10.1016/j.tcb.2006.07.004 16893648

