# Supplementary figures and images for: Translation of Cellular Protein Localization Using Convolutional Networks
Source: Front Cell Dev Biol. 2021 Aug 5;9:635231. doi: 10.3389/fcell.2021.635231 (PMC8375474; doi:10.3389/fcell.2021.635231)

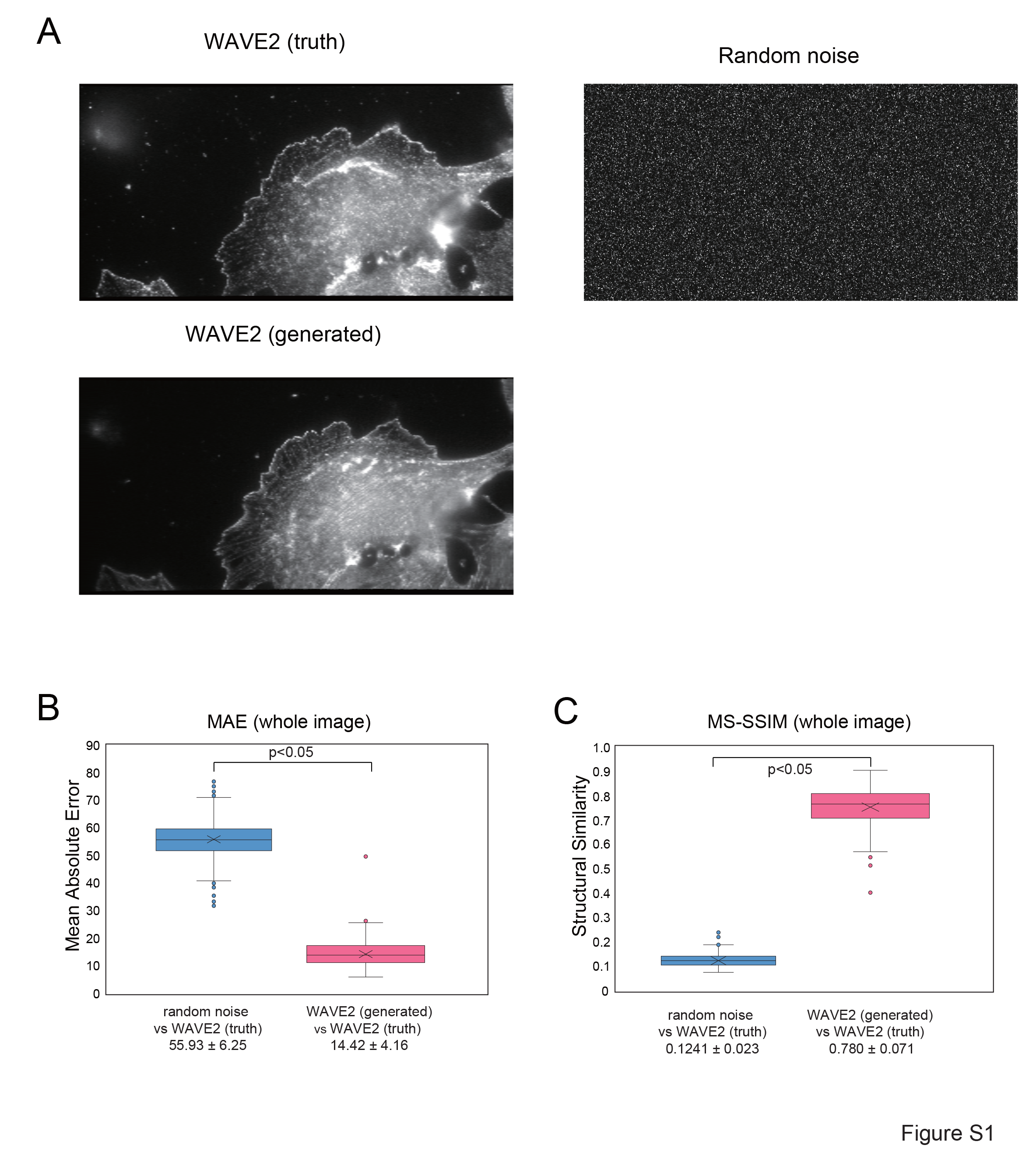

Supplement: Supplementary Figure 1 — The MAE and MS-SSIM to random noise. (A) True and the generated WAVE2 images as in Figure 2 and the random noise image that was generated by shuffling the true WAVE2 image. (B) Box plot of the MAE of entire images for panel (A) (n = 772). (C) Box plot of the MS-SSIM of entire images for panel (A) (n = 772). [file Image_1.TIF]

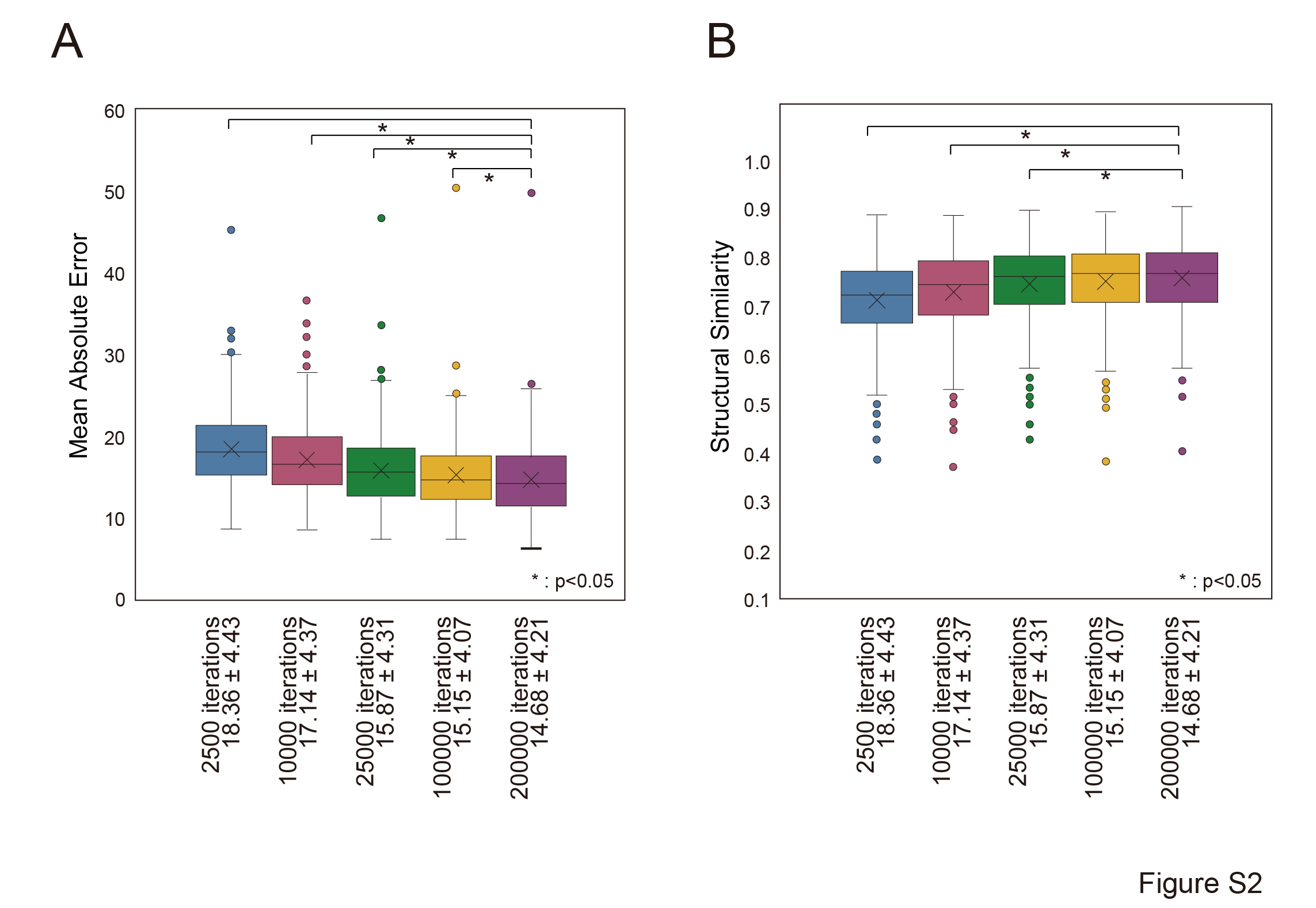

Supplement: Supplementary Figure 2 — The progress of image generation per iterations. (A) Box plot of the MAE of entire images in four-fold cross-validation for Figure 1D (n = 772). (B) Box plot of the MS-SSIM of entire images in four-fold cross-validation for Figure 1D (n = 772). Statistical significance is shown by p < 0.05 (*) by two-sample equal variance two-tailed Student’s t-test. [file Image_2.TIF]

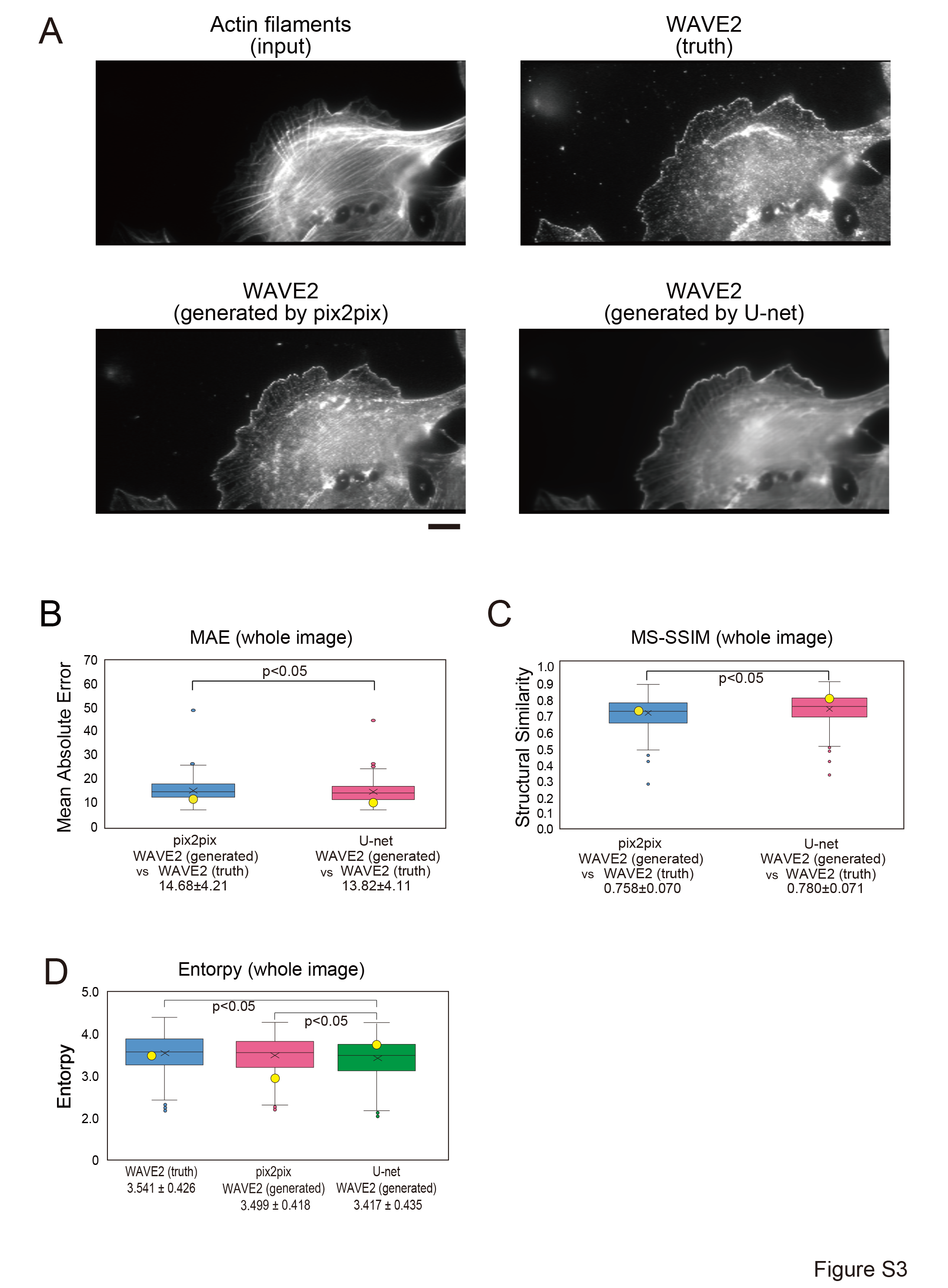

Supplement: Supplementary Figure 3 — The comparison between pix2pix and U-net. (A) Generation of a WAVE2 image by pix2pix model and U-net only model from an actin filament image. An input image (actin filament image), a ground truth image (WAVE2 immunostained image), a pix2pix output image, and a U-net only model output image. Scale bar, 10 μm. (B) Box plot of the MAE of entire images in four-fold cross-validation for panel (A) (n = 772). (C) Box plot of the MS-SSIM of entire images in four-fold cross-validation for panel (A) (n = 772). (D) Box plot of the entropy of entire images in four-fold cross-validation for panel (A) (n = 772). Statistical significance is shown by p < 0.05 by two-sample equal variance two-tailed Student’s t-test. [file Image_3.TIF]
